# Temporal and spatial dynamics of *Listeria monocytogenes* central nervous system infection in mice

**DOI:** 10.1073/pnas.2320311121

**Published:** 2024-04-18

**Authors:** Victoria Chevée, Karthik Hullahalli, Katherine G. Dailey, Leslie Güereca, Chenyu Zhang, Matthew K. Waldor, Daniel A. Portnoy

**Affiliations:** ^a^Department of Molecular and Cell Biology, University of California, Berkeley, CA 94720; ^b^Division of Infectious Diseases, Brigham and Women’s Hospital, Boston, MA 02115; ^c^Department of Microbiology, Harvard Medical School, Boston, MA 02115; ^d^HHMI, Bethesda, MD 20815; ^e^Department of Plant and Microbial Biology, University of California, Berkeley, CA 94720

**Keywords:** pathogenesis, brain, barcoding, foodborne, immunocompromised

## Abstract

*Listeria monocytogenes* is a bacterial pathogen that can cause life-threatening central nervous system (CNS) infections. While mechanisms by which *L. monocytogenes* and other pathogens traffic to the brain have been studied, a quantitative understanding of the underlying dynamics of colonization and replication within the brain is still lacking. In this study, we used barcoded *L. monocytogenes* to quantify the bottlenecks and dissemination patterns that lead to cerebral infection. Following intravenous (IV) inoculation, multiple independent invasion events seeded all parts of the CNS from the blood, however, only one clone usually became dominant in the brain. Sequential IV inoculations and intracranial inoculations suggested that clones that had a temporal advantage (i.e., seeded the CNS first), rather than a spatial advantage (i.e., invaded a particular brain region), were the main drivers of clonal dominance. In a foodborne model of cerebral infection with immunocompromised mice, rare invasion events instead led to a highly infected yet monoclonal CNS. This restrictive bottleneck likely arose from pathogen transit into the blood, rather than directly from the blood to the brain. Collectively, our findings provide a detailed quantitative understanding of the *L. monocytogenes* population dynamics that lead to CNS infection and a framework for studying the dynamics of other cerebral infections.

## Significance

*Listeria monocytogenes* is one of many pathogens that cause cerebral infections, mainly in immunocompromised individuals. In this study, we used a library of *L. monocytogenes* bacteria each labeled with a unique chromosomally encoded barcode to quantify bacterial dissemination and host bottlenecks during cerebral infection. Following intravenous inoculation, multiple clones sequentially invaded the central nervous system (CNS), but the first 1 to 3 clones that gained access to the brain became dominant. In contrast, after oral infection of immunocompromised mice, a single clone led to an overwhelming cerebral infection and profound neurological symptoms. Collectively these data provide a highly detailed map of pathogen dynamics in multiple models of cerebral listeriosis and a valuable framework to study other infections of the CNS.

Central nervous system (CNS) infections are often fatal or leave affected individuals with severe and permanent brain damage. Many microorganisms can cause CNS infections and employ a variety of mechanisms to infect the CNS. For example, the protozoan intracellular pathogen *Toxoplasma gondii* infects leukocytes that deliver the pathogen to the brain. Many viruses, including Herpes Simplex Virus-1, West Nile virus, and Rabies virus migrate within axons of the peripheral nervous system and traffic directly into the CNS. In contrast, some bacteria such as *Streptococcus pneumoniae* transcytose across the blood–brain barrier (BBB) (1). Despite understanding the routes by which certain pathogens enter the CNS, a quantitative understanding of the host bottlenecks and the patterns of pathogen colonization and replication that result in CNS infection is generally lacking.

*Listeria monocytogenes* (Lm) is a Gram-positive, facultative intracellular pathogen that can infect most mammals, including humans and ruminants. Upon ingestion of contaminated food, particularly by immunocompromised, infant, or elderly individuals, Lm can lead to sepsis and meningoencephalitis (2). Although Listeriosis is a rare disease, it has a high mortality rate (20 to 30%) (3) and is reported as the third-most lethal foodborne infection in the United States (4).

In the host, Lm can invade nonphagocytic cells or be phagocytosed, avoid autophagy, enter the cytosol, and spread from cell-to-cell using actin-based mobility (5). These abilities are all critical for the three mechanisms of cerebral infection that have been proposed: Lm can infect inflammatory monocytes (Ly6C^hi^, CCR2^pos^) in the bone marrow and hijack these migratory immune cells to traverse the BBB and invade the CNS (“Trojan horse” hypothesis) (6–9). Extracellular Lm can also directly invade endothelial cells at the BBB (10), and Lm can infect peripheral neurons and traffic along/within nerves to reach the CNS (11–16). Regardless of the precise route(s) of entry, however, a quantitative understanding of how individual Lm cells replicate and disseminate across the host during CNS infection is lacking.

Questions regarding the population dynamics of Lm within the host are challenging to answer when assessing bacterial burden alone. For example: Do only a few bacteria replicate in the brain following a rare invasion event and expand to fill the niche? Is the CNS broadly and consistently permissive to infection? Are clones from the CNS found in other tissues? Are there one or more tissues that act as reservoirs for CNS invasion? Addressing these questions will inform our understanding of pathogenesis beyond the characterization of specific virulence factors by providing a quantitative understanding of infection bottlenecks and patterns of pathogen replication and dissemination. Dissecting these underlying dynamics can provide insights into factors that promote host susceptibility and suggest avenues for therapies.

Barcoded pathogens can be used to decipher these dynamics of infection. Barcodes are unique short nucleotide sequences that generally do not impact fitness and are incorporated into the same neutral chromosomal site within each bacterium. Following deep sequencing of the barcode locus, computational methods can be used to quantify the infection bottlenecks by assessing the number of bacteria from the inoculum that give rise to the population in a tissue, known as the founding population (FP, also referred to as Ns) (17–19). In addition, comparison of barcode frequencies between organs further quantifies patterns of dissemination. Barcoded pathogens have been used to shed light on our understanding of the infection dynamics of diverse pathogens, including *Vibrio cholerae* within the gut (20), intraorgan dissemination of extraintestinal pathogenic *Escherichia coli* (21), *T. gondii* cerebral infections (22), and others (23–29). Tagged- Lm have also been used to understand the dynamics of vertical transmission between mother and fetus (30) and to elucidate bacterial trafficking circuits outside of the CNS after oral infection (31–33).

Here, we quantify the dynamics of Lm cerebral infection using a library of bacteria with ~200 barcodes and the STAMPR (Sequence Tag-based Analysis of Microbial Populations in R) analytic toolset (34). Intravenous (IV) inoculations in mice with barcoded Lm revealed that the CNS is invaded in multiple, independent waves of infection from the blood over time. Within the brain, there was marked dominance of only 1 to 3 Lm clones. Clonal dominance can be explained by timing of CNS colonization, where early arrivers become most prominent, rather than by colonization of a particularly permissive region of the brain. These dynamics were recapitulated in a model of CNS infection using foodborne Lm in streptomycin-treated immunocompromised mice. Here, bacterial replication was again substantial in the brain, although foodborne infection yielded tighter bottlenecks in all organs, and the CNS was consistently monoclonal. Collectively, our findings provide an in-depth understanding of the within-host pathogen dynamics that lead to cerebral listeriosis and a framework to study other CNS infections.

## Results

### IV Inoculation of Lm Leads to Multiple Waves of CNS Invasion.

**Fig. 1.**
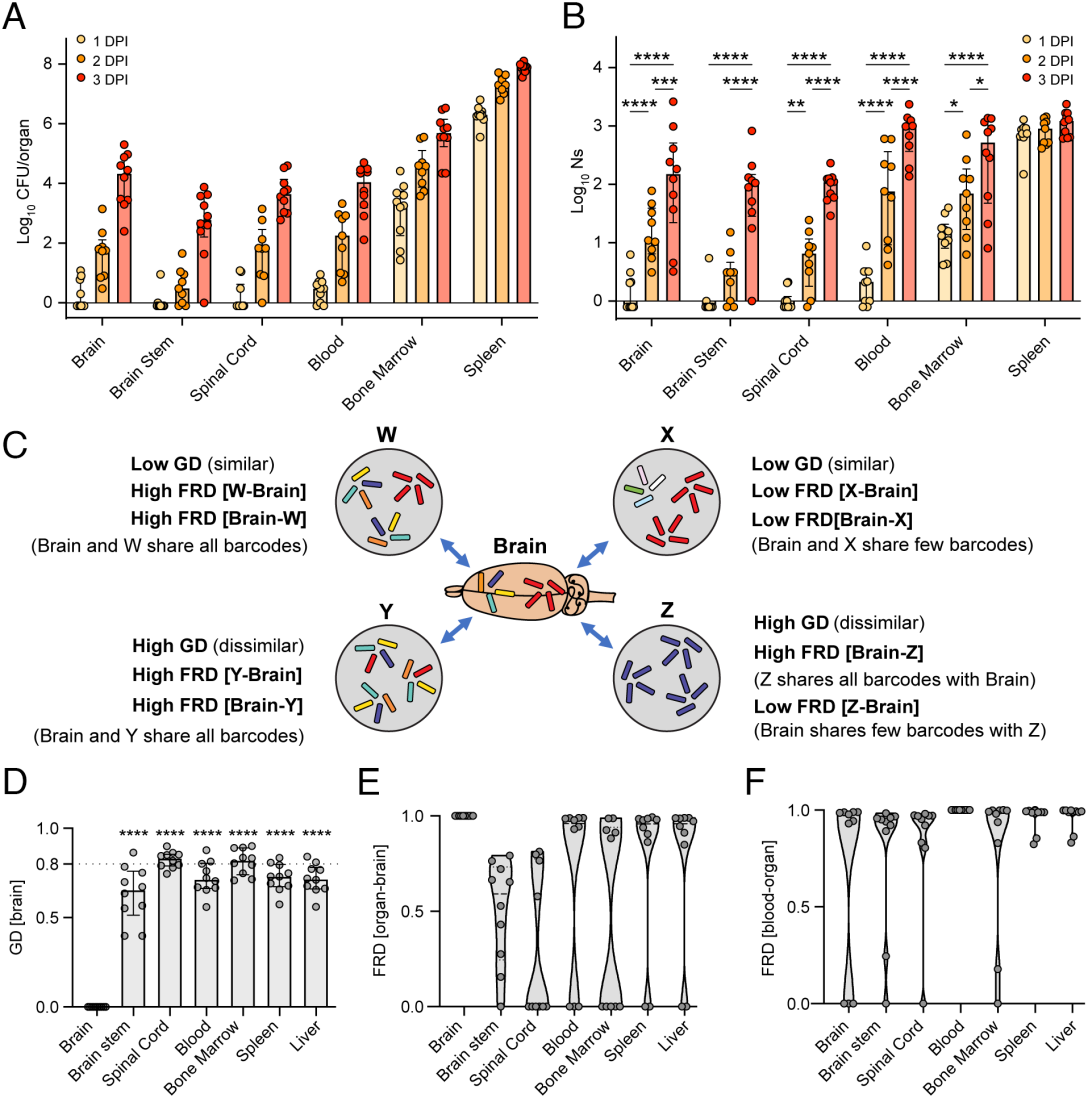
IV inoculation of *L. monocytogenes* leads to multiple waves of CNS invasion. (*A* and *B*) IV infection of C57BL/6J mice with 1 × 10^4^ CFU and collection of organs at 1, 2, and 3 DPI. Bacterial burden (CFU, *A*) and FP (Ns, *B*) per organ. Data are individual samples expressed as log-transformed values with median and interquartile range. Organs with no CFU are represented as −0.1 CFU or Ns. (*C*) Graphical depiction of the GD and the fraction of clones shared (FRD) between the bacterial populations of hypothetical organs (gray circles) and the brain. Each unique barcode is represented by a color. (*D*) GD between all organs and the brain at 3 DPI. Data are individual samples with median and interquartile range. The dotted line at 0.8 marks a “high” GD, i.e., samples that are dissimilar. (*E*) Prevalence of barcodes from the brain in other organs (FRD[organ-brain]) and (*F*) prevalence of barcodes from various organs in the blood (FRD[blood-organ]) at 3 DPI. Data are individual samples with violin plots and median. For all panels, data are combined from at least two independent experiments and 1 DPI N = 10, 2 DPI N = 9, 3 DPI N = 10. Statistical significance was calculated using two-way ANOVA for (*A*), and one-way ANOVA against the brain for (*D*): ****P < 0.0001; ***P < 0.001; **P < 0.01; *P < 0.1.

IV infections are routinely used to study murine listeriosis. In this context of early high-level bacteremia, we monitored changes in bacterial burden and barcode frequency over 3 days post infection (DPI). Mice were infected IV with a relatively low yet lethal dose (10^4^ CFU) (35) of a barcoded Lm library (31) and organs were harvested 1, 2, and 3 DPI. At 1 DPI, the Lm burden was already at ~10^6^ CFU in the spleen and close to ~10^4^ in the bone marrow, whereas the blood usually had titers that were several logs lower (~10^1^), and the CNS rarely harbored any detectable Lm. The Lm burden increased by 100- to 10,000-fold in all organs over time (Fig. 1*A*) and the increased burden in the CNS was most prominent in the brain (*SI Appendix*, Fig. S1*A*). The elevation in CNS bacterial burden over time could either be attributed to repeated influx of bacteria from other tissues within the host, or to a few bacteria that passed through a tight bottleneck and replicated within the CNS (20). These two possibilities are distinguishable by assessing the abundances of different barcodes; if additional bacteria (with new barcodes) invaded the brain, FP values would increase over time. In contrast, if the increase in CFU was due to the replication of the original invading bacteria, the number of barcodes represented in the sample would remain the same and FP would be constant. We observed an increase in FP over time (Ns, Fig. 1*B*) indicating that during the first 3 d of infection multiple Lm clones invaded the CNS and suggesting that Lm migration into the CNS, in addition to in situ CNS Lm replication, may account for the increased pathogen burden in the CNS.

### Circulating Bacteria Lead to Multiple Independent Infections of the Brain, Brainstem, and Spinal Cord.

Comparisons of barcode distributions between tissues can provide insight into routes of pathogen dissemination. The genetic distance (GD) broadly assesses the similarity between two samples: A high GD (~0.8) indicates that two samples have dissimilar populations, while a low GD indicates they have similar populations. GD is calculated based on the similarity in abundances of barcodes between two samples. However, it is possible for pairs of samples with very different barcode distributions to have the same GD value: Two similar samples that have low GD can either share many of their barcodes (Fig. 1*C*w) or share few highly abundant barcodes (Fig. 1*C*x). Likewise, two samples can have a high GD even if they share many barcodes, if there is also a marked expansion of a few barcodes in only one sample (Fig. 1*C*y). To assist in distinguishing between these possible scenarios, the STAMPR toolset also includes an additional metric known as RD (“resilient” GD) that provides insight into the contribution of dominant clones to genetic similarity (34). Quantifying the fraction of barcodes that are shared between two samples is achieved by log-normalizing the number of barcodes in each sample (FRD, “fractional” RD). For example, a high FRD_[A-B]_ value indicates that the shared barcodes between sample A and sample B represent a large fraction of the total barcodes in sample B, and a low FRD_B-A_ indicates that these same shared barcodes represent only a small fraction of the barcodes in sample A (Fig. 1*C*z). Of note, FRD is unaffected by the abundance of Lm bacteria with each barcode and is based solely on the number of barcodes shared (34).

We used GD and FRD to assess how Lm traffics into and within the CNS. The Lm clones in the brain were dissimilar to all other organs (high GD) at 3 DPI with the exception of the brainstem, where we observed moderate similarity (Fig. 1*D*). Correspondingly, FRD was moderate to low between the brain and brainstem/spinal cord (Fig. 1*E*), indicating that these CNS regions share relatively few of their barcodes with the brain. To examine whether these samples share very few barcodes simply due to poor colonization, we plotted FRD values as a function of CFU. We observed that tissues that share very few barcodes with the brain (low FRD) have a wide range of bacterial burden (*SI Appendix*, Fig. S1*B*), confirming that the brain and brainstem/spinal have distinct bacterial populations and suggesting that they are infected independently. Despite the minimal overall genetic similarity between the brain and systemic sites (blood, bone marrow, spleen, and liver, Fig. 1*D*), the FRD [systemic organ-brain] was generally high (Fig. 1*E*) regardless of CFU (*SI Appendix*, Fig. S1*C*), indicating that a large fraction of the barcodes in the brain were shared with systemic sites. The discrepancy highlighted by GD and FRD in these samples is likely due to the uneven expansion of individual clones, leading to overall dissimilar populations with underlying populations of shared barcodes (similar to Fig. 1*C*y). Notably, the fraction of barcodes shared with the blood was very high for all organs (Fig. 1*F*). Taken together, the observations of shared barcodes in the brain and blood (Fig. 1 *E* and *F*) and an increase in diversity in the brain over time (Fig. 1*B*) strongly suggest that the CNS sustains multiple waves of infection over time from Lm circulating in the blood.

### One Lm Clone Is Dominant in the Brain and Highly Abundant in the Brainstem.

**Fig. 2.**
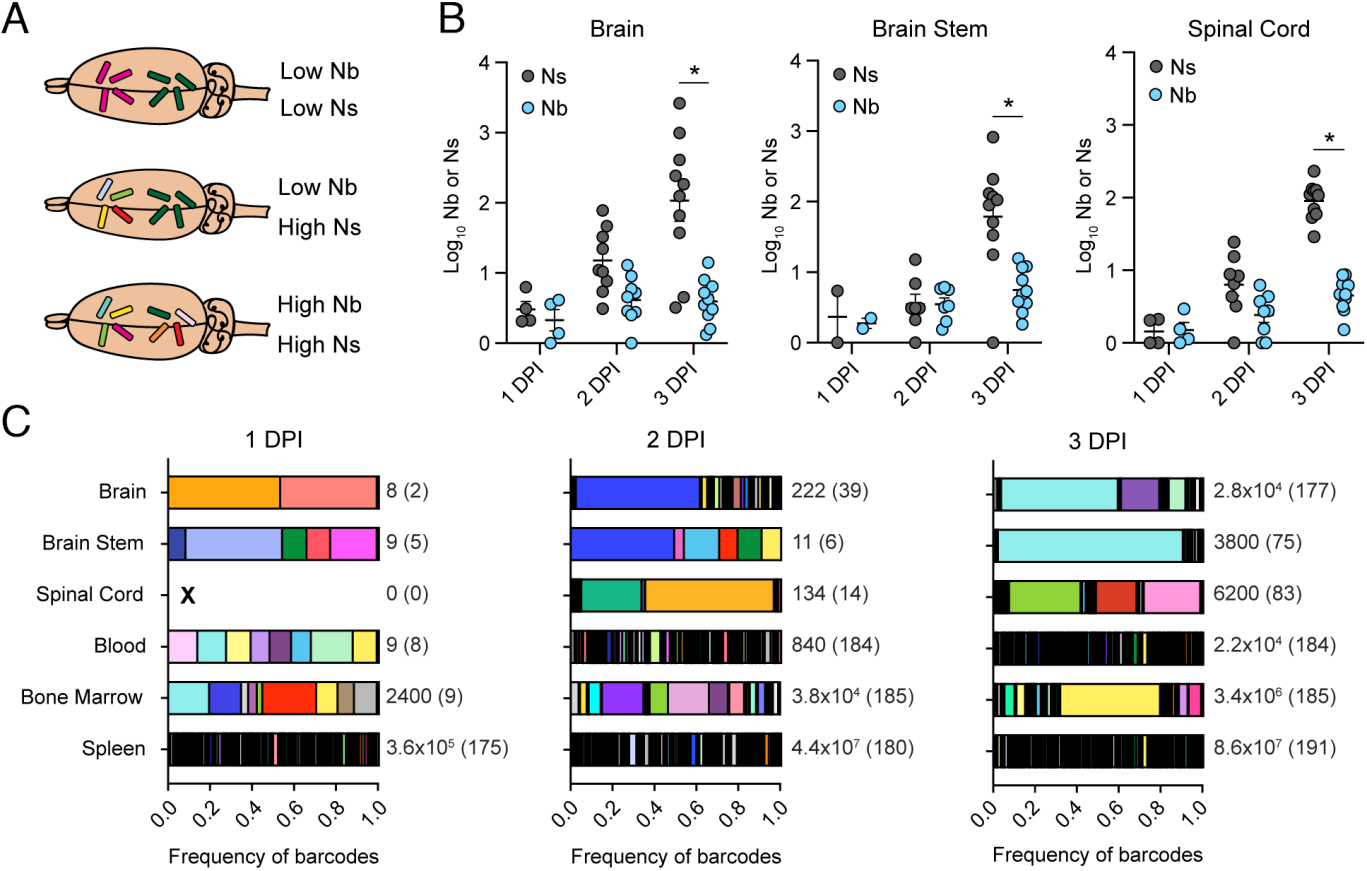
One Lm clone is dominant in the brain and highly abundant in the brainstem. (*A*) Graphical depiction of the two FP metrics: Nb and Ns, in three hypothetical brain samples. Each unique barcode is represented by a color. (*B*) Bacterial FP of the brain, brainstem, and spinal cord from the intravenously infected mice in Fig. 1. Data are log-transformed values of Nb and Ns, with mean and SEM for each group. Only samples with CFU > 0 were used: 1 DPI N = 4, 2 DPI N = 9, 3 DPI N = 10. Results are from a minimum of two independent experiments. Statistical significance was calculated using multiple Mann–Whitney tests. *P < 0.01. (*C*) Frequency of barcodes per organ for mice in Fig. 1, where each graph is one mouse representative of its group (1 DPI, 2 DPI, 3 DPI). Each color represents one barcode and the same color scheme is applied to all graphs. “X” identifies a sample with no CFU, the number besides each sample is the bacterial load (CFU), and the number in parenthesis is the number of barcodes per sample.

The observed relatively high GD (dissimilar samples) and high FRD (many clones shared) between the brain and systemic sites (Fig. 1 *D* and *E*) suggested that uneven clonal expansion may occur in the brain in situ. The presence of highly abundant barcodes can be analytically supported by comparing different metrics for estimating FP: Ns and Nb. Ns is the primary metric used to estimate FP in this work and is relatively resistant to uneven expansion of barcodes, relying instead only on their presence or absence. In contrast, Nb, another metric provided in the STAMPR toolset, determines FP by assessing differences in the frequencies of barcodes in a sample relative to the reference (which contains all ~200 barcodes). Nb is highly sensitive to uneven barcode abundances and decreases in the presence of disproportionally abundant barcodes (Fig. 2*A*). The ratio of Nb to Ns is therefore a useful metric to quantify uneven barcode distributions (Nb) relative to the overall diversity of the sample (Ns). At 1 to 2 DPI, Ns and Nb values were similar across the CNS, indicating that all bacterial clones had evenly expanded. In contrast, Ns was 50 times greater than Nb by 3 DPI, confirming the presence of disproportionately abundant clones in all parts of the CNS (Fig. 2*B* and *SI Appendix*, Fig. S1*D*).

To corroborate our findings, we assessed the frequency of individual barcodes per organ in single mice. These analyses confirmed that the blood and spleen contained diverse clones starting at 1 DPI, while diversity increased in the CNS over time. However, after 1 DPI, one clone was predominant in the brain and particularly abundant in the brainstem of most mice (Fig. 2*C* and *SI Appendix*, Fig. S1*D*). Notably, in the mice depicted in Fig. 2*B* at 2 and 3 DPI, a few clones also expanded in the spinal cord and the bone marrow but were distinct from the expanded clones in the brain and brainstem (Fig. 2*C* and *SI Appendix*, Fig. S1*D*). Together, these observations support our previous assessment that multiple seeding events account for independent infection of the brain, brainstem, and spinal cord, and additionally indicate that the bone marrow is not a continuous reservoir for Lm clones that invade the CNS as the bacterial populations do not converge over time. Furthermore, the brain and brainstem often shared an abundant barcode suggesting that this clone had either a temporal or spatial advantage to establish a proliferative niche.

### All Regions of the Brain Are Susceptible to Infection, but Lm Clones Display Heterogenous Patterns of Regional Segregation.

We hypothesized that clonal dominance in the brain was spatially driven, where a permissive niche would enable a small number of clones to expand dramatically in one location in the brain. To evaluate this hypothesis, we assessed whether bacteria were concentrated in or limited to certain regions of the brain. Mice were infected IV with the barcoded Lm library, and at 3 DPI the brain and brainstem were collected, separated across the sagittal plane (left–right), and further dissected into a total of 14 compartments (*SI Appendix*, Fig. S2*A*).

**Fig. 3.**
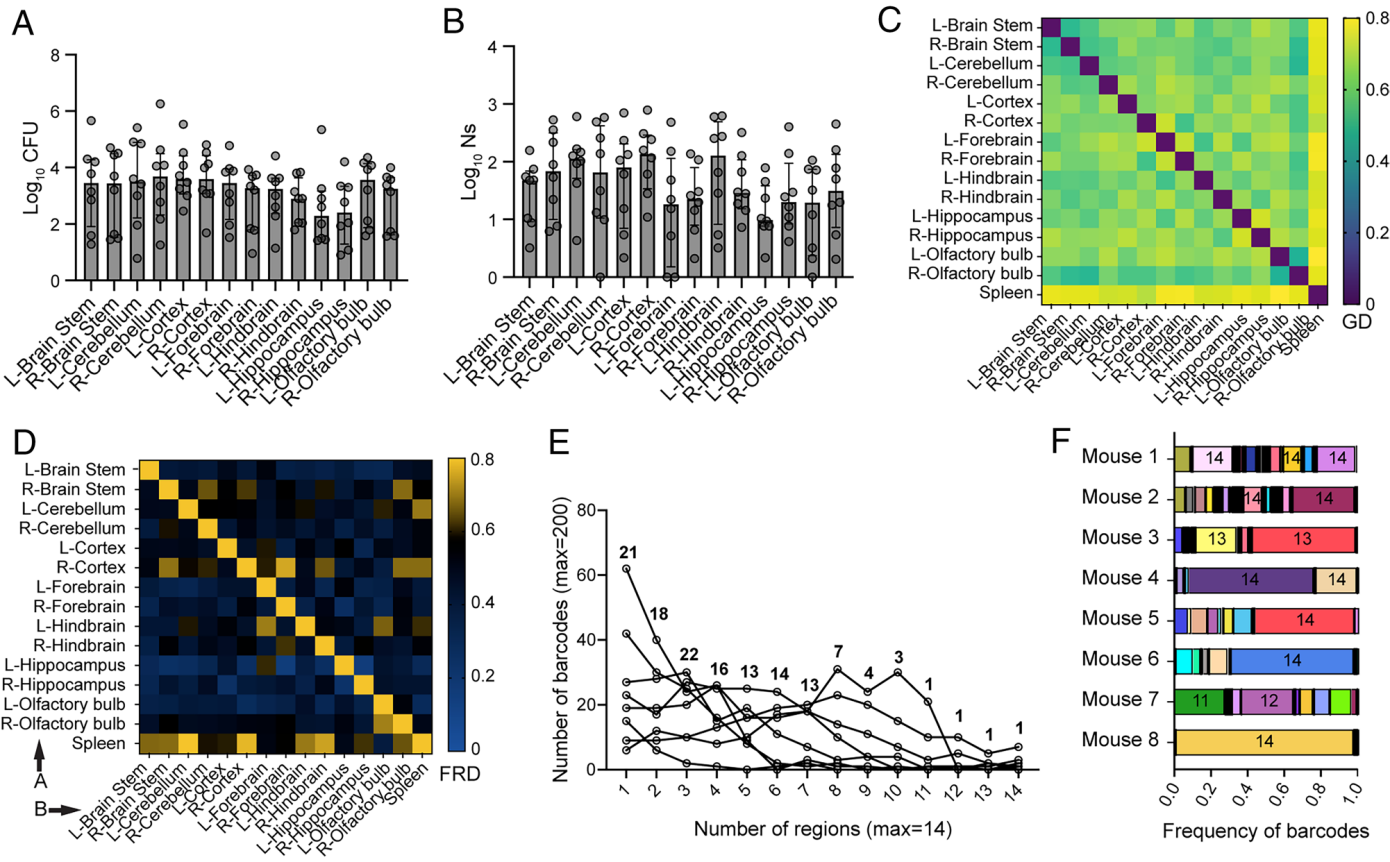
All regions of the brain are susceptible to infection, but *L. monocytogenes* clones display heterogenous patterns of regional segregation. (*A*–*F*) IV infection of C57BL/6J mice with 1 × 10^4^ CFU and collection of brain regions at 3DPI. (*A*) Bacterial burden (CFU) and (*B*) FP (Ns) per sample. Data are expressed as log-transformed values with median and interquartile range. (*C*) Similarity between each sample as assessed by GD. Data are the average value of the pairwise comparison for all mice. (*D*) Prevalence of shared barcodes (FRD) between each sample. The letters “A” and “B” identify the directionality of the measure. Data are the average value of the pairwise comparison for all mice. (*E*) Distribution of barcodes throughout the brain, as measured by the number of barcodes that are present in each fraction of brain regions, i.e., 1/14 = number of barcodes that are in only one region, 14/14 = number of barcodes that are in every region for each mouse. The line connects the data for each mouse and the numbers indicate the median number of barcodes for each region. (*F*) Frequency of barcodes within the “whole brain” of each mouse, where each row represents one mouse. Data in each row represent the combined frequency of all 14 brain regions for one mouse, normalized by the bacterial burden of each brain region in that mouse. Each color represents one barcode, and the numbers represent the number of regions this barcode was identified in (out of 14 regions). For all panels N = 8, and results are from two independent experiments.

The total bacterial burden and FPs were similar across all region (Fig. 3*A*). Thus, Lm infection was not restricted to a particular niche and all regions were capable of supporting Lm replication. Almost all compartments sampled also contained clones that were unique to that region (*SI Appendix*, Fig. S2*B*), suggesting that all regions of the brain are susceptible to invasion. GD values were intermediate (Fig. 3*C* and *SI Appendix*, Fig. S2*C*), and FRD values were consistently low (Fig. 3*D* and *SI Appendix*, Fig. S2*D*) indicating that there was modest sharing of clones between compartments. Of note, dissemination of clones between regions of the brain (moderate GD/low FRD, Fig. 3 *C* and *D*) differed from the dissemination patterns between systemic organs and the brain (high GD/high FRD, Fig. 1 *D* and *E*), suggesting that the processes that drive invasion of the brain are distinct from those that drive dissemination within the brain.

To obtain a deeper understanding of the distribution of Lm clones within the brain, we also quantified the number of regions in which each individual barcode was located. In every mouse, most barcodes were found in 1 to 7 of the 14 regions sampled, while a very small subset of clones were found throughout the entirety of the brain and brainstem (>10/14 regions, Fig. 3*E*). The spreading of dominant clones is especially apparent when examining individual mice, where 1 to 3 clones can be observed through all brain regions in each mouse (*SI Appendix*, Fig. S2*E*). Computationally combining the barcode frequencies for all the brain regions into individual “whole brain” samples while taking into account the bacterial burden of each section further confirmed that the barcodes present in most brain subsections were indeed the most abundant ones overall (Fig. 3*F*). These analyses established that clones display heterogenous regional segregation at 3 DPI; some clones disseminated, while others were locally confined. The dominance of one clone in the brain observed in Fig. 2 is thus explained by a few Lm clones efficiently spreading throughout the brain, rather than by clones accessing a specific region of the tissue that would be more permissive to invasion or expansion.

### Early Invading Lm Are the Main Drivers of Clonal Dominance in the Brain.

Since no region in the brain appeared to account for the dominance of Lm clones in the CNS, we hypothesized that clonality may be established by the timing of CNS invasion. In this model, the first clones that infect the brain would establish a niche, replicate, and spread within the tissue, while clones that invade subsequently would have a delay in colonization and thus seem more locally confined.

**Fig. 4.**
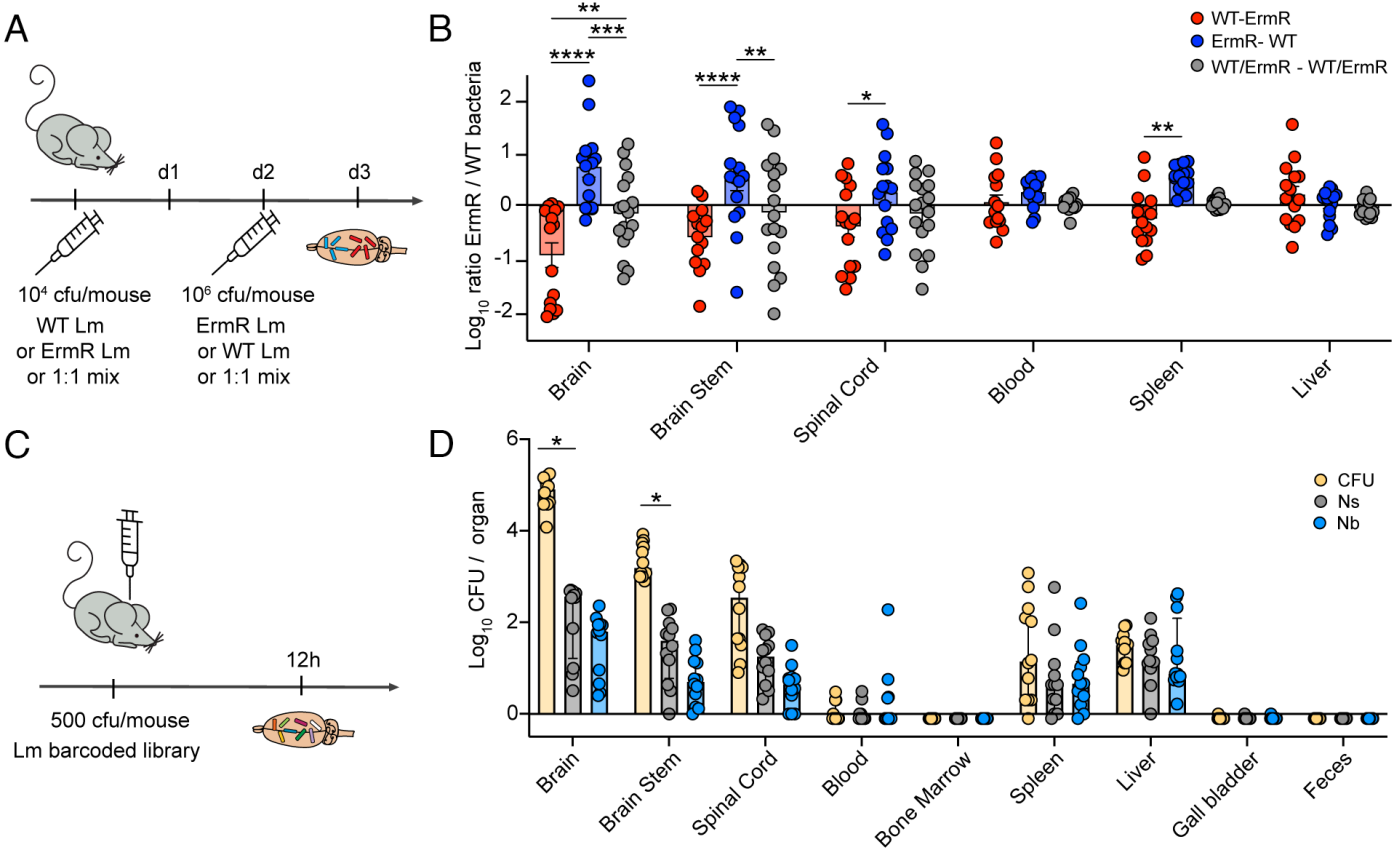
Early invading Lm are the main drivers of clonal dominance in the brain. (*A*) Graphical representation of the experimental design for sequential infections: C57BL/6J mice were inoculated intravenously with 1 × 10^4^ CFU of either WT Lm, or ErmR Lm, or a 1:1 mix of both populations. Two days later, mice were reinfected with 1 × 10^6^ CFU of the converse strain. Mice were euthanized 1 d following the second infection and the ratio of WT to ErmR Lm was assessed. (*B*) Strain composition for each sample. Results are log transformed values of the ratio of ErmR to WT bacteria and bars represent means with SEM. Statistical significance was calculated using two-way ANOVA. ****P < 0.0001; ***P < 0.001; **P < 0.01; *P < 0.1. Results are from three independent experiments with red group N = 15, blue group N = 15, gray group N = 17. (*C*) Graphical representation of the experimental design for intracranial (IC) injections: C57BL/6J mice were inoculated IC with 500 CFU of barcoded Lm. Samples were collected for sequencing 12 h post injection. (*D*) Bacterial burden (CFU) and FP metrics (Ns and Nb) following IC inoculation. Results are log-transformed values with median and interquartile range. Results are from three independent experiments for a total of N = 12. Samples with no CFU are represented as −0.1. Statistical significance was calculated using multiple Mann–Whitney tests between CFU and Ns, and between Ns and Nb. *P < 0.01.

To assess whether the timing of CNS invasion is the main driver of clonal dominance in the brain, we experimentally imposed an order of invasion using a simplified system of tagged Lm. Mice were infected IV with 10^4^ CFU of either wild-type (WT) or erythromycin-resistant (ErmR) bacteria. At 2 DPI, the animals were given a second IV dose with 10^6^ CFU of the other Lm strain. A control group received a 1:1 dose of each strain in both infection rounds. The 10^6^ CFU second dose corresponds to the bacterial burden in the liver at 2 DPI (*SI Appendix*, Fig. S1*E*) which is where 90% of the inoculum is found 30 min post IV infection (35). This dose was chosen to ensure it would neither be so low as to be immediately cleared by the already activated immune system, nor so high to cause the mice to immediately succumb to the infection. At 3 DPI the organs were collected, and the proportion of each strain was assessed per sample (Fig. 4*A*).

In the brain and brainstem, we observed a significant bias toward greater abundance of bacteria from the first inoculum. In the control group, where both strains were inoculated together, no bias was observed, confirming that the strains have equivalent fitness. In agreement with the barcoded Lm analyses, which revealed that the spinal cord populations were distinct from those of the brain and brainstem, the spinal cord samples were highly variable and less impacted by the timing of inoculation, suggesting that independent factors affect spinal cord and brain/brainstem population shifts. Similarly, neither strain was more abundant in the liver, blood, or spleen when compared to the control group, suggesting that infection of these organs is mostly unaffected by the timing of invasion. Notably, the bacterial population in the brain and brainstem samples were not entirely from the initial infection group, but rather a mix of bacteria from both waves of infection with a strong bias toward the first group (Fig. 4*B*). This suggests that the first invasion event does not prevent further infection by subsequent Lm waves. Together these observations suggested that timing provides an opportunity for “early arrivers” to become prevalent in the brain/brainstem.

To further probe the role of timing in the establishment of clonal dominance in the brain/brainstem, we bypassed all barriers to invasion and infected mice directly into the brain (Fig. 4*C*). Following inoculation of 500 CFUs, the brain burden exceeded those observed with IV inoculation, reaching 10^5^ CFU by 12 HPI (Fig. 4*D* vs. Fig. 1*A*). Ns was ~10^2^ (Fig. 4*D*), which is similar to the inoculum size, indicating a wide bottleneck to cerebral colonization and an ability to replicate in situ. Although bacterial burden dramatically increased during these first 12 h, there was no difference between Ns and Nb in the brain (Fig. 4*D*) indicating that replication was relatively even across the population. Thus, during intracranial infection, when timing is removed as a variable, the Lm clones expand 1,000-fold in the brain (CFU = 10^5^, Ns = 10^2^), but do so relatively evenly. Together these data further support timing as a critical factor that controls clonal dominance in the brain during cerebral listeriosis.

### Food-Borne Infection of Immunocompromised Mice Reproducibly Leads to Cerebral Listeriosis.

**Fig. 5.**
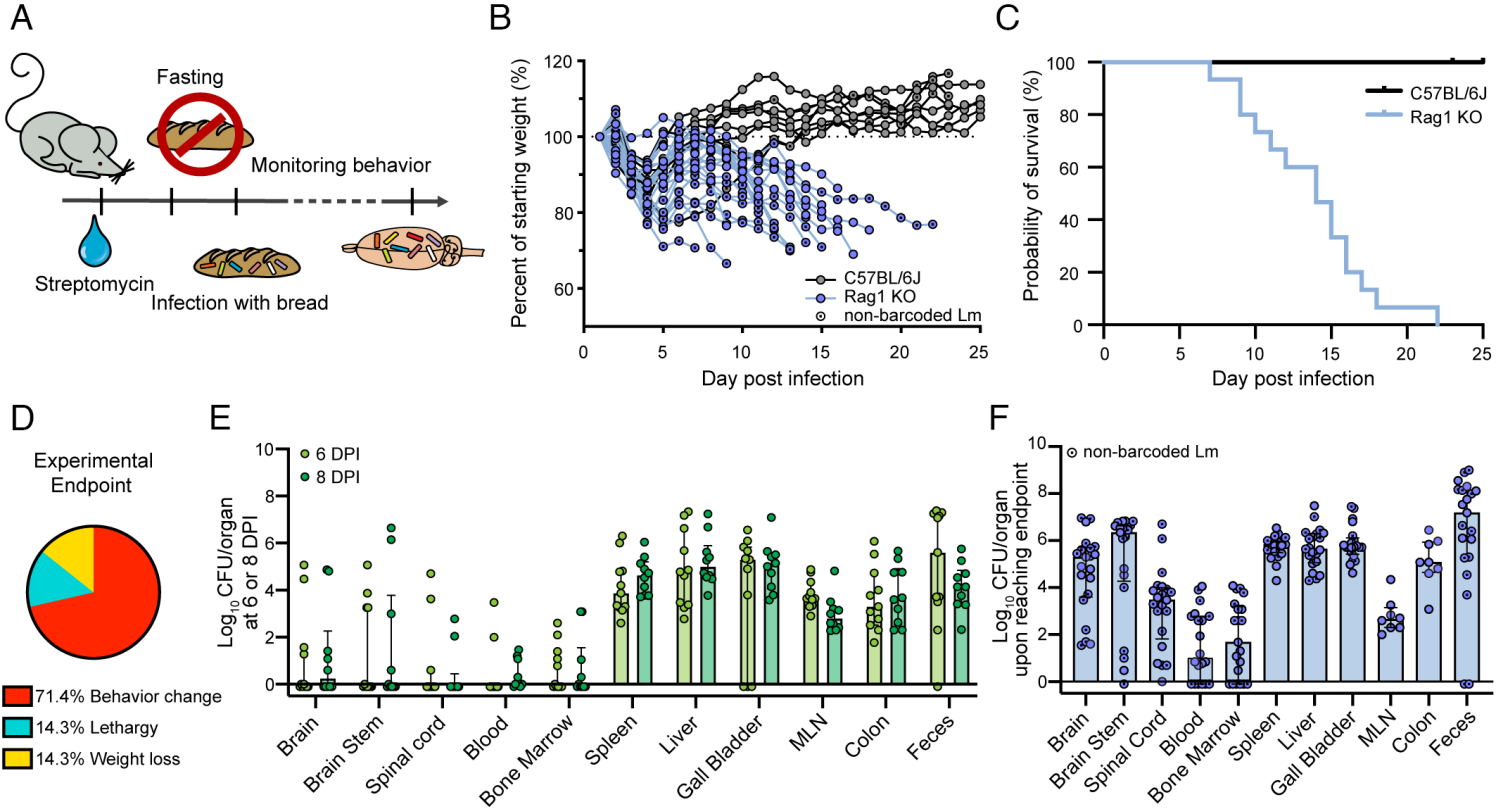
Food-borne infection of immunocompromised mice reproducibly leads to cerebral listeriosis. (*A*) Graphical representation of the experimental design for oral infections: Mice were treated with streptomycin (20 mg/mL) in the drinking water for 48 h, fasted for 24 h, then infected with 1 × 10^9^ CFU of either barcoded or nonbarcoded Lm and monitored daily for behavioral changes and body weight loss. (*B*) Change in weight over time for Rag1 KO mice and C57BL/6J controls. Results are expressed as the percentage of body weight compared to 1 DPI and each line represents one mouse. Mice that received nonbarcoded Lm are marked with a black dot and the dashed line indicates 100%. (*C*) Survival rate for Rag1 KO and C57BL/6J mice. (*D*) Experimental endpoint for Rag1 KO mice, where “behavior change” refers to circling behavior, head tilt, or mild to severe paralysis, “lethargy” refers to lack of activity, and “weight loss” refers to a loss of 30% of original weight. (*E*) Bacterial burden at “early” times of infection with barcoded Lm (6 DPI or 8 DPI) for Rag1 KO mice. Results are expressed as log-transformed values with median and interquartile range. (*F*) Bacterial burden at “late” times of infection (upon reaching experimental endpoint criteria) for Rag1 KO mice. Results are expressed as log-transformed values with median and interquartile range and mice that received nonbarcoded Lm are marked with a black dot. Data for (*B*–*D* and *F*) are combined from three independent experiments with Rag1 KO N = 21 (including barcoded Lm N = 8) and C57BL/6J N = 8. Data for (*E*) are combined from four independent experiments with 6 DPI N = 11 and 8 DPI N = 10.

Most studies of cerebral listeriosis, including our work presented thus far, are based on nonnatural routes of infection, including IV (6, 8–10), intraperitoneal (7, 8), intramuscular (13), intragastric (9, 14), intranasal (14), and intracranial (9). These methods are valuable and allow us to probe specific aspects of the infection process, but bypass many of the host barriers that ordinarily constrain Lm dissemination and restrict expansion during natural infections. We adapted foodborne infection models (36, 37) with immunocompromised mice, in order to study the dynamics of severe CNS infection following a more natural inoculation. In this model, Rag1-deficient mice (Rag1 KO), which lack B and T cells, were treated with oral streptomycin for 2 d, fasted for ~20 h, then inoculated by consumption of bread contaminated with 10^9^ Lm CFU (Fig. 5*A*). Of note, sequence tag-based analysis of microbial populations (STAMP) barcodes are integrated at a fitness neutral site in the Lm chromosome and are expected to have little to no effect on bacterial physiology or pathogenesis (20, 31), so oral infections were performed with either barcoded or nonbarcoded Lm and all mice were pooled together for Fig. 5.

During the first 5 d postingestion of infected food, Rag1 KO mice lost up to 25% of their original weight followed by a phase of recovery, similar to their WT counterparts. Within a week most WT mice had recovered, and none succumbed to infection. In marked contrast, most Rag1 KO mice began losing weight again by 7 DPI and every animal reached an experimental endpoint criterium by 30 DPI, although the exact timing of disease progression varied between mice (Fig. 5 *B* and *C*). The experimental endpoint was variable, but ~70% of mice developed behavioral changes tied to cerebral infection such as circling (Movie S1), head tilt, or paralysis (Fig. 5*D*). Thus foodborne infection of Rag1 KO mice leads to disease outcomes similar to those exhibited by cerebral listeriosis-afflicted ruminants (38).

Changes in behavior are manifestations of brain damage (39) suggesting that Lm crosses the BBB long before clinical signs become evident. To further characterize “early” stages of infection, the Lm burden both within and outside the CNS was determined at 6 or 8 DPI. Bacterial burdens in the CNS were highly variable (Fig. 5*E*), consistent with the variable rates of disease progression, and were generally lower than the burden found in other organs. At “late” times of infection, when mice reached the experimental endpoint criteria described in Fig. 5*D*, the median bacterial burden in the CNS remained variable, but was generally highest in the brainstem (median ~10^6^ CFU) and lowest in the spinal cord (median ~10^4^ CFU, Fig. 5*F* and *SI Appendix*, Fig. S3*C*). Even though mice were euthanized at different DPI, the bacterial burden in the spleen and liver was less variable with a median of ~10^6^ CFU and the blood and bone marrow were generally poorly infected at this late time point (Fig. 5*F*). Overall, these observations reveal that low bacterial burden at systemic sites, when compared to IV infection (Fig. 1*A*), still leads to consistent cerebral infection.

### Dynamics of Food-Borne Lm Invasion of the CNS Differ from the IV Model.

**Fig. 6.**
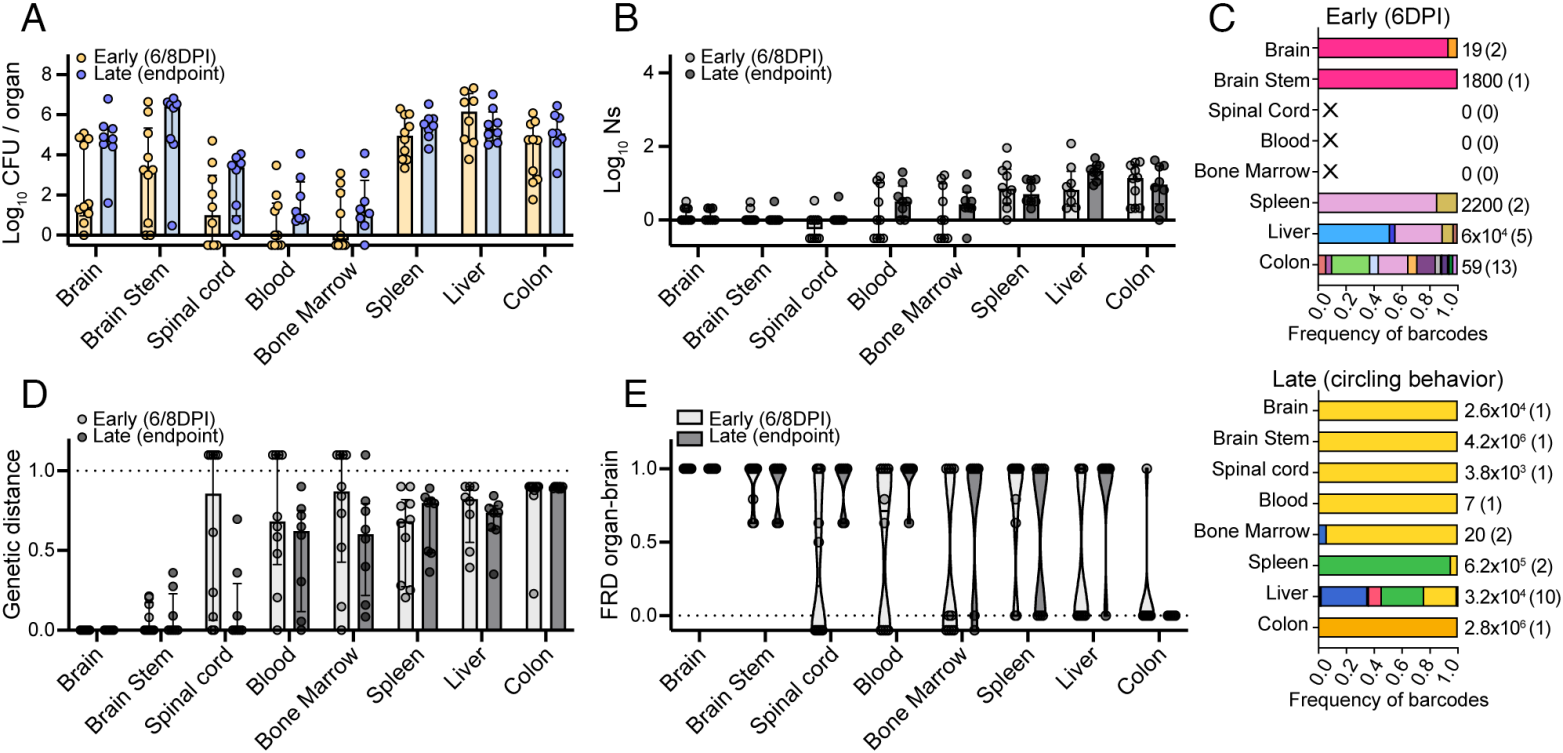
Dynamics of food-borne Lm invasion of the CNS differ from the IV model. (*A*) Bacterial burden (CFU) and (*B*) FP (Ns) for the mice in Fig. 5 *E* and *F* that were infected with barcoded Lm. “Early” refers to samples collected at 6 or 8 DPI, but only from mice where brain CFU > 0. “Late” refers to samples collected when mice reached experimental endpoint criteria as described in Fig. 5*D*. Results are log-transformed values with median and interquartile range. Organs with no CFU are represented as −0.5. (*C*) Frequency of barcodes for representative mice of the “early” and “late” groups. Each color represents one barcode and the same color scheme was applied to both graphs. “X” represents a sample with no CFU, the number besides each sample is the bacterial load, and the number in parenthesis is the number of barcodes per sample. (*D*) Genetic distance (GD) to brain. Data are individual samples with median and interquartile range and samples with no CFU are represented as 1.1. (*E*) Prevalence of barcodes from the brain in other organs (FRD[organ-brain]). Data are individual samples with violin plots and median and samples with no CFU are represented as −0.1. Data for the “early” group are combined from four independent experiments (subset of mice from Fig. 5*E*) with N = 8. Data for the “late” group are combined from two independent experiments (subset of mice from Fig. 5*F*, which received barcoded Lm) N = 8.

We used STAMPR-based metrics to determine whether host bottlenecks and Lm dissemination patterns were similar in the foodborne and IV models of cerebral listeriosis. Mice were compared across multiple infections with barcoded Lm (with equivalent inoculums, *SI Appendix*, Fig. S3*D*) where “early” samples were collected at 6- or 8 DPI from mice that have brain CFU > 0 (subset of mice from Fig. 5*E*) and “late” samples were collected from mice upon reaching endpoint criteria (subset of mice from Fig. 5*F*). Since mice progress through infection at different rates (Fig. 5 *B* and *C*), bacterial burden for “early” mice was variable, but overall we found that the Lm burden in the CNS increased by ~4logs from early to late times of infection (Fig. 6*A*) while FP sizes remained low over time (Fig. 6*B*). Ns from orally infected mice was generally lower at all sites than during IV infections (Figs. 1*B* and 6*B*) suggesting that passage through the intestinal barrier in mice lacking Rag1 comprises a very stringent bottleneck. Notably, in the CNS, Ns was generally ~1 in both early and late time points, suggesting a mostly monoclonal CNS (Fig. 6 *B* and *C* and *SI Appendix*, Fig. S3 *A* and *B*). Thus, in marked contrast to the IV model where there is continued Lm invasion of the CNS over the 3-d infection period (Fig. 1*B*), increase in bacterial burden in the oral model is likely primarily driven by replication within rather than migration into the CNS. In addition, the total bacterial burden and FP in the spleen, liver, blood, and bone marrow remained lower in oral infections compared to IV infections (Figs. 1 *A* and *B* and 6 *A* and *B* and *SI Appendix*, Fig. S1*F*), suggesting that systemic sites are overall more restrictive to Lm colonization in the oral model compared to IV.

At early times of infection, the Lm populations in the CNS were similar to each other (low GD and high FRD[organ-brain]), except the spinal cord which was often uninfected (Fig. 6 *D* and *E*). At any time, the brain only ever harbored 1 to 3 clones and these barcodes were generally shared with the rest of the CNS (Fig. 6*C* and *SI Appendix*, Fig. S3 *A* and *B*). The clones in the brain were however consistently underrepresented in the colon (Fig. 6*E*) and the colon population was always highly dissimilar to the brain (GD, Fig. 6*D*), suggesting that Lm does not primarily depend on retrograde trafficking from peripheral neurons that innervate the gut to infect the brain. Taken together, our findings reveal that the dynamics of Lm CNS invasion in the oral and IV models differ; during oral infection in Rag KO mice, only a very small number of Lm clones invade the CNS and subsequently replicate locally.

## Discussion

In this study, we used barcoded Lm and leveraged STAMPR analytics to uncover the patterns of pathogen trafficking, replication dynamics, and host bottlenecks in multiple models of CNS infection. Following IV inoculation, Lm infects the CNS in multiple waves, independently invading the brain, brainstem, and spinal cord (Fig. 1 *A*–*E*). All regions of the CNS shared most of their barcode diversity with the blood (Fig. 1*F*), establishing the blood as the most probable reservoir for continuous CNS invasion. Nevertheless, the brain and brainstem harbored a few highly expanded clones (Fig. 2). Clonal dominance was not due to a spatial advantage (Fig. 3), but the predominant clones instead had a temporal advantage in the brain, where the first Lm to invade became more prominent (Fig. 4). During foodborne infection in immunocompromised mice, we observed extremely tight bottlenecks, and the CNS was nearly monoclonal although robustly colonized (Figs. 5 and 6). Together, our findings quantify the contribution of several barriers that Lm must cross to infect the CNS and reveal the ability of a single clone to cause cerebral infection, highlighting the remarkable power of using barcoded bacteria to quantify pathogen dissemination during infection.

The use of barcoded Lm has previously been applied to models of systemic and foodborne infection, and our results build on these prior studies by mapping the dynamics of Lm invasion of the CNS. Zhang et al. demonstrated that Lm can escape tissue containment and enter circulation from highly infected systemic organs, thus promoting pathogen exchange between the spleen and liver (31). Our study reveals that pathogen exchange in systemic organs also facilitates continual invasion of the CNS. These results also mirror the findings of Wincott et al. (22), who used barcodes to study the neurotropic parasite *T. gondii* and found that the BBB imparted limited restraint to CNS infection. Correspondingly, following Lm oral infection in Rag KO mice, the spleen and liver harbored fewer bacteria, the blood had a low burden, and the CNS remained monoclonal. These observations are consistent with the idea that a CFU threshold in systemic sites must be met to enable robust entry of Lm into circulation and allow continuous invasion of the brain. During oral infection, a failure to reach this threshold limits the ability of Lm to escape systemic organs, reducing bloodborne bacteria, and ultimately leading to a monoclonal CNS.

Multiple mechanisms have been proposed to explain how Lm first invades the CNS. The most accepted model proposes that Lm hijacks bone-marrow-derived monocytes to reach the BBB. Although these cells are very efficient at promoting brain infection (6) and CCR2^−/−^ mice show a decreased brain bacterial burden (9), the Lm populations in the brain and bone marrow did not converge over time during IV infection (Fig. 2*C* and *SI Appendix*, Fig. S1*D*), suggesting that the dominant clones in the bone marrow do not continuously and robustly invade the CNS. However, in our study, we collected only femurs, and it remains possible that other bone marrow sites harbor clones shared with the CNS, particularly the skull marrow (40). Defining the heterogeneity of Lm across bone marrow sites could be facilitated by STAMPR in future studies. An alternative model proposed that Lm traffics within neurons via the peripheral nervous system (11–16). This hypothesis is particularly attractive since the gastrointestinal tract is densely innervated (41) and harbors large numbers of Lm following oral infection (31, 32). However, we did not observe similar bacterial populations between the colon and CNS (Fig. 6). It is possible that intraneural trafficking originates from other tissues, but our findings thus far argue against a direct gut-brain axis as the principal route for continuous pathogen trafficking into the CNS. Even though each of the proposed routes could be responsible for the first invasion event, our data support the blood as the most likely source of bacteria that invade the brain over time.

We found that timing of CNS invasion drives clonal dominance in the brain, where clones that first invade the brain are more abundant than clones that arrive in subsequent waves. The dominance of the first organisms to arrive in a new niche is known as the “priority effect” and has been previously described in the study of complex microbial populations, including several models of intestinal colonization (42, 43). However, it is highly likely that the mechanisms that drive priority effects during intestinal colonization are distinct from those that drive priority effects during CNS invasion. Notably, direct antagonism between bacteria and competition for nutrients play major roles in priority effects in intestinal colonization by extracellular bacteria (43, 44). Since Lm is an intracellular pathogen and there is limited evidence for the presence of other abundant microbes in the brain, we hypothesize that neither plays a major role in this context. We propose that the priority effect in the Lm-infected CNS is a consequence from the fact that the first clones to invade the brain have more time to replicate before the host succumbs to infection.

Our study further emphasizes the impact of the infection route in studies of pathogen dissemination, since the pathogen dynamics during foodborne infection were distinct from the dynamics during IV infection. To mimic a more natural CNS infection, we adapted a model of foodborne infection where mice were pretreated with streptomycin (36). Other models of oral cerebral listeriosis have previously been developed with no antibiotic treatment and/or the use of clinical isolates (9, 37, 45, 46). Antibiotic pretreatment promotes dysbiosis which limits colonization resistance in the gut and facilitates a more efficient infection (32, 36). Dysbiosis is also a common occurrence in immunocompromised individuals who are most at risk of cerebral listeriosis (47) and defining the role of the microbiota in Lm CNS invasion will be worthy of further study. The use of Rag1 KO mice enables infection in a context where pathogen clearance is not achieved. Indeed, CD8^+^ T cells are required for clearance of Lm from a host (48, 49), and SCID mice, which have limited B and T cells, become chronically infected with Lm (50). Some bacterial factors are also known to facilitate CNS invasion (10) and many neurotropic clinical isolates have been identified (9, 45). Quantifying the specific contribution of host and bacterial factors on pathogen dissemination to the CNS will be facilitated by the use of barcoded Lm and provide substantially greater insight into the principles that underlie Lm CNS infection in nature. Overall, our work provides insights into the dissemination patterns of intracellular bacteria to the brain, laying the groundwork for barcode-based analysis of CNS invasion with other neurotropic pathogens.

## Material and Methods

### Animal Models.

C57BL/6J mice were purchased from the Jackson Laboratory (stock #000664) and maintained under specific pathogen-free conditions at the University of California, Berkeley animal facility. Rag1 KO mice (B6.129S7-Rag1tm1Mom/J, Jackson Laboratories stock #003729) were obtained from the Barton lab at the University of California, Berkeley, and maintained under specific pathogen-free conditions within the University of California, Berkeley animal facility. Only 8- to 12-wk-old female mice were used for all experiments with sex- and age-matched controls according to institutional guidelines for animal care. This study was performed in strict accordance with the recommendations in the Guide for the Care and Use of Laboratory Animals of the National Research Council of the National Academy of Sciences and with university regulations. All protocols were reviewed and approved by the Animal Care and Use Committee at the University of California, Berkeley (AUP-2016-05-8811).

### Bacterial Strains and Growth Conditions.

All *L. monocytogenes* strains used in this study were derived from 10403S (51) which is streptomycin resistant and propagated in filter-sterilized brain heart infusion (BHI) medium (BD) at 37 °C while shaking with streptomycin (200 μg/mL) overnight. Cell density was measured by optical density (OD600). Inoculums were prepared by washing log-phase bacteria twice in PBS and resuspending in the appropriate volume of PBS depending on the infection route. Frozen bacterial stocks were stored at –80 °C in BHI plus 25% glycerol.

### IV Infections.

Mice were infected intravenously via the tail vein with 10^4^ CFU of *L. monocytogenes* strains in 200 µL of sterile PBS as previously described (52). Briefly, 1, 2, or 3 DPI, mice were euthanized, and tissues were harvested as described below. For sequential infections, mice were infected intravenously via the tail vein with 10^4^ CFUs of *L. monocytogenes* in 200 µL of sterile PBS. Forty-eight hours following the first injection, mice were injected again intravenously via the tail vein with 10^6^ CFU of Lm in 200 µL of sterile PBS. Twenty-four hours following the second infection, mice were euthanized, and tissues were harvested as described below.

### Intracranial Infections.

Mice were infected intracranially with 500 CFU of *L. monocytogenes* strains with 5 µL of sterile PBS as previously described (53). Briefly, mice were anesthetized with 2% isoflurane, given pre-emptive analgesics (Buprenorphine 0.1 mg/kg and Meloxicam 10 mg/kg), and arranged on Angle-two stereotactic frame (Leica, Nussloch, Germany). The incision area was swabbed with three alternating wipes of 70% ethanol and betadine scrub with sterile applicators prior to performing minimally damaging craniotomies. The stereotaxic surgery coordinates used for targeting the striatum, relative to bregma, were 2 mm anterioposterior, 1 mm mediolateral, 1.5 mm dorsoventral. Infusion of *L. monocytoge*nes (500 CFU) was performed with a syringe to deliver 0.5 µL per minute for a total volume of 5 µL. Postinfusion, the syringes were left in position for 2 min before slow removal from the injection site, which was then cleaned, sutured, and surgically glued. Throughout the procedure, mice were kept at 37 °C for warmth and Puralube Vet Ointment (Dechra, NDC #17033-211-38, Northwich, England) was applied to the outside of the eyes. Mice were euthanized and tissues were harvested 12 h post infection.

### Foodborne Infections.

Prior to infection, 20 mg/mL of streptomycin sulfate was added to the drinking water. After 24 h, chow was removed to initiate an overnight fast and mice were transferred to fresh cages. 48 h after streptomycin was added to the water, mice were isolated, fed a 3-mm piece of bread with 3 µL of butter and 10^9^ CFU *L. monocytogenes* in 5 µL PBS. Mice were then returned to cages containing standard drinking water and chow. The inoculum was plated for CFU to check the efficiency of each infection. Fecal pellets were collected at 1 DPI for each mouse and only animals with 1 DPI CFU/gram > 10^6.5^ were kept for further analysis. Mice were monitored daily for weight loss and behavioral changes including head tilt, circling, mild to severe paralysis, and lethargy. Upon reaching an endpoint criterium as described on our animal use protocol (any of the behaviors described in Fig. 5: behavior change, lethargy, or 30% weight loss), mice were euthanized, and tissues were harvested.

### Tissue Harvest.

Blood (~500 µL) was collected via cardiac puncture in 100 µL 50 mM EDTA as an anticoagulant [adapted from Senay et al. (45)]. Immediately after blood collection, mice were perfused with 20 mL PBS. Most samples were homogenized in 0.1% IGEPAL CA-630 (Sigma) and for bone marrow, the left femur and tibia were collected together and then crushed with a mortar and pestle before homogenizing. Gallbladders and feces were homogenized in 1×PBS. Colons were cut longitudinally, washed with PBS, and incubated in RPMI (Gibco) containing 5% fetal calf serum, HEPES, L-glutamine, and 100 μg/mL gentamicin for 45 min at 37 °C. This procedure kills all extracellular bacteria within the tissue. Colons were then washed six times to remove all gentamicin by placing the tissue into 10 mL of fresh PBS on a rotator at 4 °C for 20 min before homogenizing in 1 mL 0.1% IGEPAL. Clean autoclavable probes were used and changed for the homogenization of each sample to avoid cross-contamination. Dilutions of homogenates were plated to enumerate CFU and full samples were plated for STAMPR analysis following incubation at 37 °C overnight. In instances where growth of commensal bacteria was a concern, plates were supplemented with nalidixic acid, LiCl, and glycine, which does not affect Lm growth. Antibiotics and media supplements were used at the following concentrations: streptomycin at 200 μg/mL, nalidixic acid at 15 μg/mL, LiCl at 6 mg/mL, and glycine at 6 mg/mL.

### STAMP.

STAMP was performed as previously described (20, 34). The library with ~200 barcodes, which was created by A. Louie in 2019 (32), was freshly inoculated into BHI broth from frozen stocks and grown to log-phase for each experiment. Inoculum samples were collected frequently to ensure that the population structure remained stable across all experiments. To process samples, Lm colonies were washed off plates, collected in PBS with 25% glycerol, diluted in water, and boiled for 1 h at 80 °C. The barcode-containing region was amplified from the genome using custom forward and reverse primers (*SI Appendix*, Table S1) with OneTaq HS Quick-Load Master Mix (New England Biolabs) on boiled samples. Samples were amplified using primers containing TruSeq indexes and adapters for Illumina sequencing. PCR products were pooled and purified using the GeneJet PCR purification kit. Purified products were sequenced on either an Illumina MiSeq or Illumina NextSeq 1000. Reads were demultiplexed using a custom R script and trimmed and mapped to a list of ~200 barcodes using Qiagen CLC Genomics Workbench with default settings. Mapped reads were exported as a CSV table. FP estimates were determined using the STAMPR analysis toolset (34). GD was estimated with Cavalli-Sforza chord distance (54) as previously described (20). FP (Ns) sometimes were computed as <1 which is biologically aberrant so Nb < 1 values were manually amended to Nb = 1. Sequencing data and original script have been deposited on GitHub (https://github.com/hullahalli/stampr_rtisan) and are publicly available. Any additional information required to reanalyze the data reported in this paper is available from the lead contact upon request.

### Statistical Analysis.

All statistical analyses were performed using GraphPad Prism version 10.0.1 for MacOS, GraphPad Software, San Diego, CA. Details for all statistical analyses can be found in the figure legends.

## Supplementary Material

Appendix 01 (PDF)

Movie S1. **Mouse with circling behavior following oral infection** Recording of an orally infected Rag1 KO mouse exhibiting circling behavior 12 days post infection. This mouse received streptomycin pre-treatment and was infected with 1x10^9^ CFU of barcoded *L. monocytogenes*.

## Data Availability

Sequencing data and original script data have been deposited in Github: https://github.com/hullahalli/stampr_rtisan (55).
