# Supplementary material for: Temporal and spatial dynamics of Listeria monocytogenes central nervous system infection in mice
Source: Proc Natl Acad Sci U S A. 2024 Apr 18;121(17):e2320311121. doi: 10.1073/pnas.2320311121 (PMC11046682; doi:10.1073/pnas.2320311121)
Supplement: Supplementary file 1 — Appendix 01 (PDF) [file pnas.2320311121.sapp.pdf]

PNAS

Supporting Information for

Temporal and spatial dynamics of *Listeria monocytogenes* central nervous system infection in mice

Victoria Chevée, Karthik Hullahalli, Katherine G. Dailey, Leslie Güereca, Chenyu Zhang,

Matthew K. Waldor, Daniel A. Portnoy

Correspondence: portnoy@berkeley.edu (D.A.P.), Hullahalli@g.harvard.edu (K.H.)

This PDF file includes:

Figures S1 to S3
Table S1
Legend for Movie S1

Other supporting materials for this manuscript include the following:

Movie S1

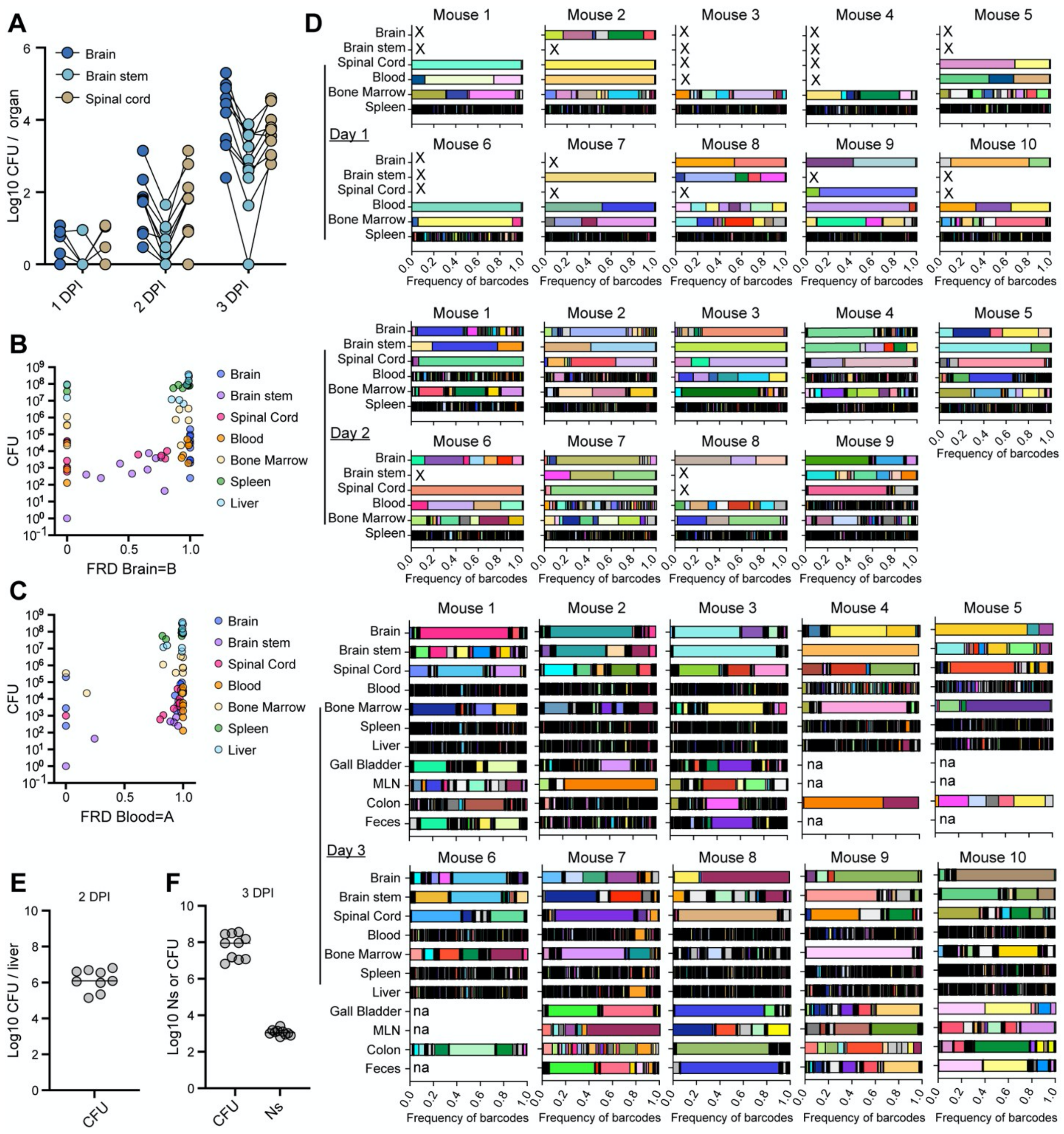

Supplementary Figure S1: Intravenous infections of C57BL/6J mice intravenously infected with $1x10^4$ CFU of *L. monocytogenes*. *(A)* Bacterial burden (CFU) in the central nervous system over 3 days post infection (DPI). Results are expressed as log-transformed CFU per organ and each line connects the samples from one mouse. *(B-C)* Comparison between bacteria burden and FRD[organ-brain] *(B)* or FRD[blood-organ] *(C)* for all mice at 3 DPI. Each dot represents one sample and is colored by the tissue type. *(D)* Frequency of barcodes per organ over 3 days post infection, where each graph represents one mouse, and each color represents one barcode (the same color scheme was applied to the entire panel). Data for *A-D* were collected from the same mice as Figure 1 and Figure 2. (E) Bacterial burden in the liver at 2 DPI. Results are expressed as log-transformed CFU with median. *(F)* Bacterial burden and founding population (Ns) in the liver at 3 DPI. Results are expressed as log-transformed values with median. Data for *E and F* are combined from at least two independent experiments each with 2 DPI n=9, 3 DPI n=10.

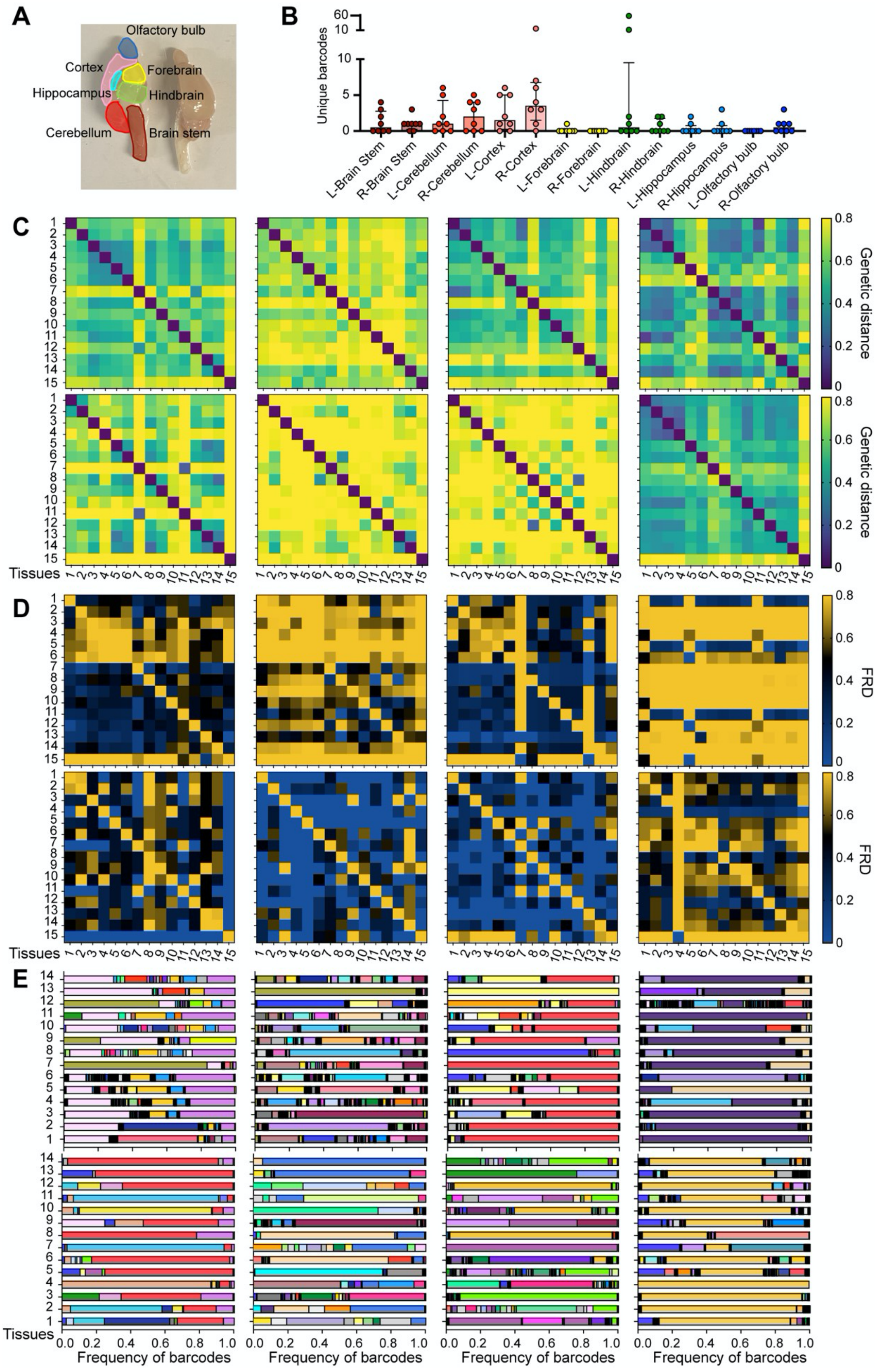
A
Olfactory bulb
Cortex
Forebrain
Hippocampus
Hindbrain
Cerebellum
Brain stem
B
Unique barcodes
L-Brain Stem
R-Brain Stem
L-Cerebellum
R-Cerebellum
L-Cortex
R-Cortex
L-Forebrain
R-Forebrain
L-Hindbrain
R-Hindbrain
L-Hippocampus
R-Hippocampus
L-Olfactory bulb
R-Olfactory bulb
C
Genetic distance
Tissues
D
FRD
E
Frequency of barcodes

Supplementary Figure S2: Dissection of brain regions 3 days post intravenous infection. *(A)* Depiction of the regions of the brains and brain stems collected 3 days post intravenous infection of C57BL/6J mice with $1x10^4$ CFU of *L. monocytogenes*. Brains were dissected into left and right hemispheres, then each further separated into the following 7 regions: olfactory bulbs, cortex, hippocampus, cerebellum, brain stem (medulla and pons only), and the remaining area was separated halfway along the coronal plane into two sections labeled “forebrain” and “hindbrain” (includes midbrain). *(B)* Sites of entry into the brain. Data represent the number of clones that are uniquely identified in only one brain region for each mouse, with median and interquartile range. *(C)* Genetic distance between samples at 3 days post IV infection. Each heatmap represents one mouse with a threshold for dissimilarity set at 0.8. *(D)* Fraction of shared barcodes (FRD) between samples at 3 days post IV infection. Each heatmap represents 1 mouse with a threshold for dissimilarity set at 0.8. *(E)* Frequency of tags at 3 days post IV infection. Each graph represents one mouse and each color represents one barcode (the same color scheme was applied to the entire panel). For *A-E* all data were collected from the same mice as Figure 3. For panels *C-E* the tissues are as follows: 1- left brain stem, 2- right brain stem, 3- left cerebellum, 4- right cerebellum, 5- left cortex, 6- right cortex, 7- left forebrain, 8-right forebrain, 9- left hindbrain, 10- right hindbrain, 11- left hippocampus, 12- right hippocampus, 13- left olfactory bulb, 14- right olfactory bulb, 15- spleen.

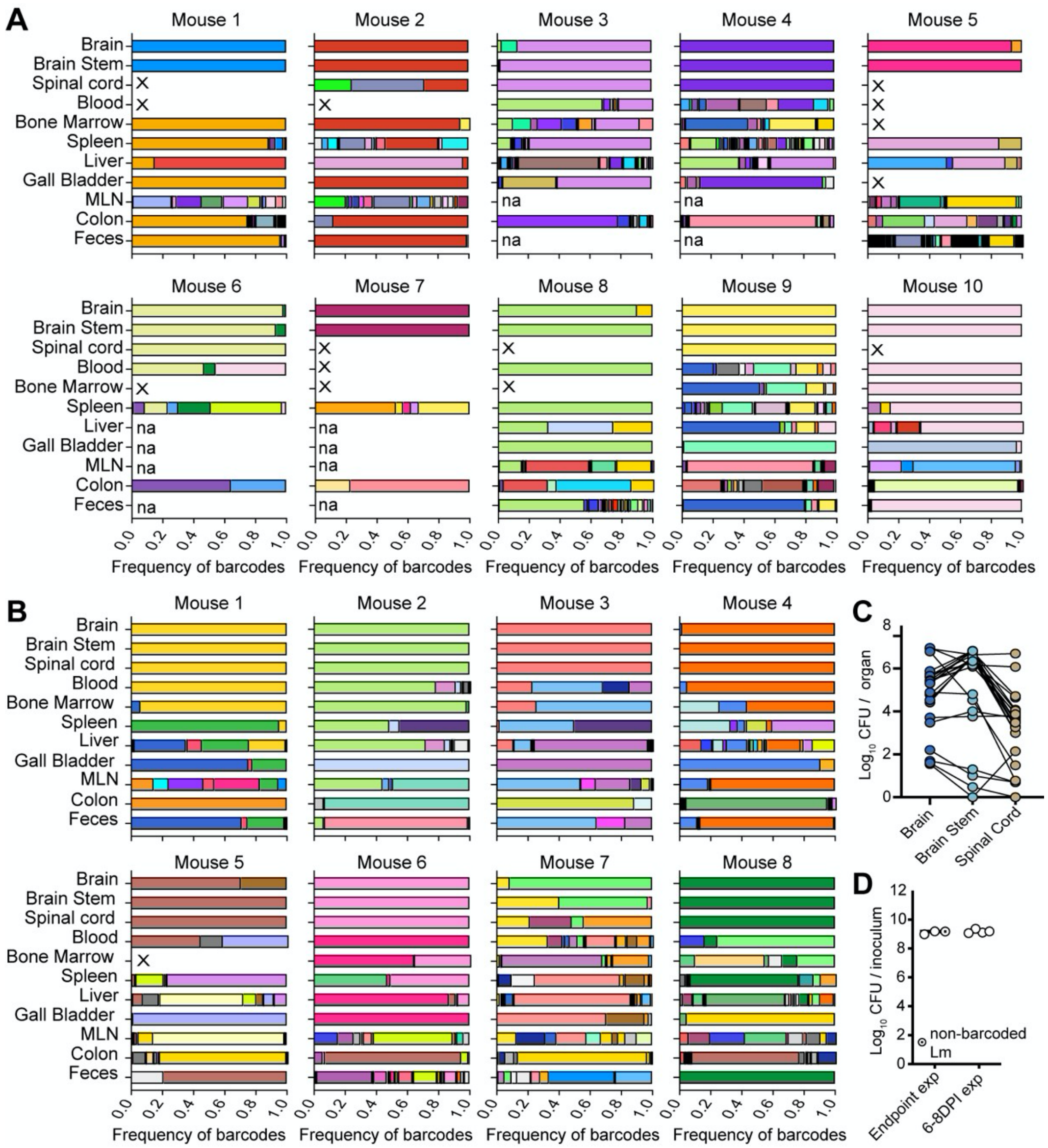

Supplementary Figure S3: Oral infection of mice with 1x10^{9} CFU of *L. monocytogenes.* *(A-B)* Frequency of tags for Rag1 KO mice sacrificed at 6-8 DPI (*A*) or upon reaching endpoint criteria (*B*). Each graph represents one mouse, and each color represents one barcode (the same color scheme is applied to all graphs). "X" identifies a sample with no CFU and "na" means the sample was not collected. *(C)* Bacterial burden (CFU) in the central nervous system of Rag1 KO mice upon reaching endpoint criteria, where each line connects the samples of one mouse. (D) Inoculum for experiments where mice were collected upon reaching endpoint criteria (Fig. B-D,F and Fig. 6) or at 6-8 DPI (Fig. 5E and Fig. 6). Each dot represents the inoculum for one experiment. Results for (C-D) are expressed as log-transformed values.

Oligo Name	Sequence
Var13	AATGATACGGCGACCACCGAGATCTACACTCTTTCCCTACACGACGCTCTTCCG ATCTTACTTGTAAAACGACGGCCAGT
Var14	AATGATACGGCGACCACCGAGATCTACACTCTTTCCCTACACGACGCTCTTCCG ATCTGCTATTGTAAAACGACGGCCAGT
Var15	AATGATACGGCGACCACCGAGATCTACACTCTTTCCCTACACGACGCTCTTCCG ATCTCGACTGTTGTAAAACGACGGCCAGT
Var16	AATGATACGGCGACCACCGAGATCTACACTCTTTCCCTACACGACGCTCTTCCG ATCTTAGCGTATTGTAAAACGACGGCCAGT
Var17	AATGATACGGCGACCACCGAGATCTACACTCTTTCCCTACACGACGCTCTTCCG ATCTTGCTGAGTCTTGTAAAACGACGGCCAGT
Var18	AATGATACGGCGACCACCGAGATCTACACTCTTTCCCTACACGACGCTCTTCCG ATCTGCTGATAGTATTGTAAAACGACGGCCAGT
Var19	AATGATACGGCGACCACCGAGATCTACACTCTTTCCCTACACGACGCTCTTCCG ATCTCAACTGCGTAGCTTGTAAAACGACGGCCAGT
Var20	AATGATACGGCGACCACCGAGATCTACACTCTTTCCCTACACGACGCTCTTCCG ATCTATGACCGTCTCGATTGTAAAACGACGGCCAGT
AD002	CAAGCAGAAGACGGCATACGAGATACATCGGTGACTGGAGTTCAGACGTGTGC TCTTCCGATCTTGTCTCATGAGCGGATACA
AD004	CAAGCAGAAGACGGCATACGAGATTGGTCAGTGACTGGAGTTCAGACGTGTGC TCTTCCGATCTTGTCTCATGAGCGGATACA
AD005	CAAGCAGAAGACGGCATACGAGATCACTGTGTGACTGGAGTTCAGACGTGTGC TCTTCCGATCTTGTCTCATGAGCGGATACA
AD006	CAAGCAGAAGACGGCATACGAGATATTGGCGTGACTGGAGTTCAGACGTGTGC TCTTCCGATCTTGTCTCATGAGCGGATACA
AD007	CAAGCAGAAGACGGCATACGAGATGATCTGGTGACTGGAGTTCAGACGTGTGC TCTTCCGATCTTGTCTCATGAGCGGATACA
AD012	CAAGCAGAAGACGGCATACGAGATTACAAGGTGACTGGAGTTCAGACGTGTGC TCTTCCGATCTTGTCTCATGAGCGGATACA
AD013	CAAGCAGAAGACGGCATACGAGATTTGACTGTGACTGGAGTTCAGACGTGTGC TCTTCCGATCTTGTCTCATGAGCGGATACA
AD014	CAAGCAGAAGACGGCATACGAGATGGAACTGTGACTGGAGTTCAGACGTGTGC TCTTCCGATCTTGTCTCATGAGCGGATACA

Supplementary Table S1: Oligonucleotides for sequencing

Primers with names beginning with “var” are forward primers and primers with names beginning with “ad” are reverse primers.

Supplementary Movie S1: Mouse with circling behavior following oral infection

Recording of an orally infected Rag1 KO mouse exhibiting circling behavior 12 days post infection. This mouse received streptomycin pre-treatment and was infected with $1x10^9$ CFU of barcoded *L. monocytogenes*.
